# Body weight status, eating behavior, sensitivity to reward/punishment, and gender: relationships and interdependencies

**DOI:** 10.3389/fpsyg.2014.01073

**Published:** 2014-10-20

**Authors:** Anja Dietrich, Martin Federbusch, Claudia Grellmann, Arno Villringer, Annette Horstmann

**Affiliations:** ^1^Department of Neurology, Max Planck Institute for Human Cognitive and Brain SciencesLeipzig, Germany; ^2^IFB Adiposity Diseases, Leipzig University Medical CenterLeipzig, Germany; ^3^Clinic for Cognitive Neurology, University Hospital LeipzigLeipzig, Germany; ^4^Mind and Brain Institute, Berlin School of Mind and Brain, Humboldt-University and CharitéBerlin, Germany; ^5^Collaborative Research Center 1052A1, University of LeipzigLeipzig, Germany; ^6^Collaborative Research Center 1052A5, University of LeipzigLeipzig, Germany

**Keywords:** eating behavior, gender differences, obesity, personality traits, reward sensitivity, punishment sensitivity, Behavioral Activation System, Behavioral Inhibition System

## Abstract

Behavioral and personality characteristics are factors that may jointly regulate body weight. This study explored the relationship between body mass index (BMI) and self-reported behavioral and personality measures. These measures included eating behavior (based on the *Three-Factor Eating Questionnaire*; Stunkard and Messick, 1985), sensitivity to reward and punishment (based on the *Behavioral Inhibition System/Behavioral Activation System* (*BIS/BAS) scales*) (Carver and White, 1994) and self-reported impulsivity (based on the *Barratt Impulsiveness Scale-11*; Patton et al., 1995). We found an inverted U-shaped relationship between restrained eating and BMI. This relationship was moderated by the level of disinhibited eating. Independent of eating behavior, *BIS* and *BAS* responsiveness were associated with BMI in a gender-specific manner with negative relationships for men and positive relationships for women. Together, eating behavior and *BIS/BAS* responsiveness accounted for a substantial proportion of BMI variance (men: ∼25%, women: ∼32%). A direct relationship between self-reported impulsivity and BMI was not observed. In summary, our results demonstrate a system of linear and non-linear relationships between the investigated factors and BMI. Moreover, body weight status was not only associated with eating behavior (*cognitive restraint* and *disinhibition*), but also with personality factors not inherently related to an eating context (*BIS/BAS*). Importantly, these relationships differ between men and women.

## INTRODUCTION

Body weight regulation and the development of obesity are associated with multiple interdependent factors and mechanisms. These mechanisms include, at the individual level, genetic and endocrine factors as well as behavioral and personality characteristics (e.g., Williamson et al., 1995; Bellisle et al., 2004; Provencher et al., 2004; Dina et al., 2007; Farooqi et al., 2007; Frayling et al., 2007; Klok et al., 2007; Ahima, 2008; Davis and Fox, 2008; Rosenbaum et al., 2008; Page et al., 2011). One of the most important factors contributing to body weight status is eating behavior, which is commonly assessed by the *Three-Factor Eating Questionnaire* (*TFEQ*; Stunkard and Messick, 1985). The *TFEQ* measures three dimensions of eating behavior: *cognitive restraint (CR)*, *disinhibition (DIS)*, and *susceptibility to hunger* or *hunger (HUN)*, for short. *Cognitive restraint* measures individual control over eating. Restrained eaters attempt to suppress impulses to eat in order to pursue long-term weight goals. Typical characteristics are avoidance of fattening foods and eating of small portions. The factor *disinhibition* reflects overeating tendencies. Disinhibited eaters typically initiate eating because of external environmental cues, such as palatable food. They have difficulties resisting food stimulation and/or eat under emotional distress. Considering this, *cognitive restraint* (conscious restriction of food intake) and *disinhibition* (tendency to overeat) conceptually represent antagonistic concepts. The third factor, *hunger*, characterizes the extent to which hunger feelings are experienced and evoke food intake. While *hunger* and *disinhibition* are positively associated with body mass index (BMI; e.g., Bond et al., 2001; Boschi et al., 2001; Bellisle et al., 2004; Bryant et al., 2008; Lesdéma et al., 2012), the relationship of *cognitive restraint* and BMI seems to be more complex and non-linear: In normal weight individuals they are usually positively associated, but the relationship is typically negative in overweight and obese individuals (e.g., Foster et al., 1998; Lluch et al., 2000; Bellisle et al., 2004; Provencher et al., 2004; de Lauzon-Guillain et al., 2006; Cappelleri et al., 2009). Additionally, *cognitive restraint* and *disinhibition* are not independently related to BMI, they interactively influence body weight status (Stunkard and Messick, 1985; Westenhoefer et al., 1990; Williamson et al., 1995; Hays et al., 2002; Dykes et al., 2004). Specifically, *cognitive restraint* attenuates the effect of *disinhibition* on BMI. What is more, previous investigations indicate that eating behavior (including presumably also underlying biological mechanisms) and body weight status mutually influence each other. For example, there are alterations in the level of *cognitive restraint* as well as *disinhibition* in response to dieting (e.g., Karlsson et al., 1994; Pekkarinen et al., 1996; Foster et al., 1998; Westerterp-Plantenga et al., 1998; Dalle Grave et al., 2009; Savage et al., 2009; Tucker and Bates, 2009).

In addition to eating behavior, various personality traits are related to food consumption and weight status (Faith et al., 2001; Elfhag and Morey, 2008). One of the most popular models of personality that may explain individual variations in food intake is the *reinforcement sensitivity theory* (RST; Gray, 1970, 1982, 1987; Gray and McNaughton, 2000). Based on this theory, two general motivational systems that underlie behavior and affect have been suggested—the Behavioral Inhibition System (BIS) and the Behavioral Activation System (BAS), commonly assessed by the *BIS/BAS* scales (Carver and White, 1994). The BIS represents the aversive motivational system. It is sensitive to signals of punishment, reward omission, and novelty. The BIS is supposed to inhibit behavior that may lead to negative or painful outcomes and is associated with negative affect (negative reinforcement). The BAS reflects the appetitive motivational system. It is sensitive to signals of reward and the avoidance of punishment (positive reinforcement). High BAS responsiveness is related to enhanced approach behavior and positive affect.

As food can be both a positive or negative reinforcer, responsiveness of these systems potentially plays a substantial role in body weight regulation. However, the relationship between sensitivity to reward (as a facet of BAS responsiveness) and BMI has been almost exclusively investigated in women. Investigations showed positive associations of reward sensitivity with BMI and eating habits supporting weight gain (Davis et al., 2004, 2007; Franken and Muris, 2005). In addition, reward responsiveness has been related to neural responses. In particular sensitivity to reward was shown to be positively associated with neural responses to pictures of highly palatable food in a fronto-striatal-amygdala network (Beaver et al., 2006). Further findings indicate that long-lasting overeating and obesity account for adaptations of the reward system (Wang et al., 2001; Volkow et al., 2008; de Weijer et al., 2011). In combination with the aforementioned findings, these studies led to the development of a *hyper- vs*. *hyposensitivity theory* of reward in obesity (e.g., Davis and Fox, 2008). According to this theory, some individuals show an inherent heightened reward sensitivity (*hypersensitivity*) and are particularly susceptible to the rewarding properties of high-calorie food. They are thus supposed to regularly overeat on fattening food and consequently become overweight or obese. Prolonged overeating and corresponding obesity, on the other hand, are associated with alterations in the dopaminergic (DA) reward circuitry, presumably to compensate for an enhanced DA tone (Wang et al., 2001; Volkow et al., 2008; de Weijer et al., 2011). These alterations are assumed to result in *hyposensitivity* to reward in obese individuals as well as in increased hedonic eating to compensate this deficiency. This theory was explored by Davis and Fox (2008). According to their model, in both genders BMI and sensitivity to reward are non-linearly associated by an inverted U-shaped relationship. More specifically, the authors reported high reward sensitivity in overweight and mildly obese participants and low reward sensitivity in morbidly obese ones. Thus, although sensitivity to reward and sensitivity to punishment are assumed to be dispositional traits rather than transient states or symptoms (Wilksch and Wade, 2009), at least sensitivity to reward seems to be flexible to a certain extent.

To our knowledge, the association between sensitivity to punishment and BMI so far has not yet been studied directly, although several studies demonstrate a relationship between sensitivity to punishment and eating disorders. Similar to obese subjects, patients suffering from bulimia nervosa and anorexia nervosa (binge/purge subtype) are characterized by overeating. This points at possible similarities in the underlying personality structure leading to a shared decision-making profile (Brogan et al., 2010). Studies investigating eating disorders repeatedly report high punishment responsiveness in patients compared to healthy controls (e.g., Harrison et al., 2010, 2011). In addition, sensitivity to punishment has been shown to be positively associated with symptoms of binge eating (Davis, 2013). Again, these studies are almost exclusively restricted to women. Matton et al. (2013) clustered adolescents with respect to reward and punishment responsiveness. Interestingly, the cluster of subjects with both high reward sensitivity and high punishment sensitivity outscored other clusters on self-reported eating problems (i.e., data regarding concerns about eating, body shape and weight as well as emotional and external eating). Although girls were more likely to belong to this cluster, effects were similar for both girls and boys. Based on these findings, Matton et al. (2013) proposed that adolescents in this cluster are especially vulnerable to the development of eating problems.

Sensitivity to reward is regarded as one aspect of the multidimensional psychological construct *impulsivity* (e.g., Guerrieri et al., 2008). Generally, impulsive behavior is rapid and rash, characterized by a lack of planning and less forethought about consequences of spontaneous actions (Moeller et al., 2001). As the term “multidimensionality” indicates, impulsivity covers several different but related concepts. The relationship to overeating is thus not straightforward. While individual differences in some aspects of impulsivity are likely to contribute to the ability to resist overeating, others may not. Various tasks that assess aspects of impulsive behavior indicate altered decision-making in overweight and obese individuals. In *Delay Discounting Tasks* or *Delay Gratification Paradigms*, for example, obese subjects in general (Rasmussen et al., 2010) or obese women in particular (Weller et al., 2008; Weygandt et al., 2013) chose more often immediate but smaller monetary or food-related reward in comparison to normal weight control subjects. In the *Iowa Gambling Task* obese volunteers preferred high immediate reward despite long-term losses. This was shown in both genders (Pignatti et al., 2006; Brogan et al., 2011), women (Horstmann et al., 2011), or men (Koritzky et al., 2012). In addition, obese women and children of both genders lacked appropriate inhibitory control in the non-reward related *Stop Signal Task* (Nederkoorn et al., 2006a,b). Another task measuring inhibitory control, the *Go/No-Go Task*, showed especially overweight and obese adolescent girls to have difficulties inhibiting prepotent motor responses to high-calorie food (Batterink et al., 2010). Heightened impulsivity was also reported for overweight children (Braet et al., 2007) as well as overweight and obese adults (e.g., Chalmers et al., 1990; Mobbs et al., 2010) based on different self-reported measures. For example, Mobbs et al. (2010) reported higher levels of urgency, lack of perseverance and strong sensitivity to reward in overweight and obese women. They concluded that overweight and obesity are associated with problems in inhibiting dominant behavior and intrusive thoughts. Within the obese population, there is evidence for heightened self-reported impulsivity among severely compared to less severely obese individuals (Rydén et al., 2003), and impulsivity was further related to higher food intake in women using the *Barratt Impulsiveness Scale* (BIS; Guerrieri et al., 2007).

An important factor that contributes to differences in eating behavior and personality, and probably also to body weight regulation, is gender. Women, for example, have higher scores of *cognitive restraint* and *disinhibition* compared to men (Bellisle et al., 2004; Provencher et al., 2004; Li et al., 2012). Additionally, eating disorder symptomatology is more prevalent among women (e.g., Keel et al., 2007; Matton et al., 2013; Yean et al., 2013). Furthermore, men and women differ in personality traits such as impulsivity. For example, higher sensation seeking and behavioral risk taking was observed in men compared to women (Arnett, 1992; Byrnes et al., 1999; Cross et al., 2011). Additionally, both gender-independent and gender-specific effects have been reported, for example, with respect to the *Iowa Gambling Task* and weight status (Pignatti et al., 2006; Brogan et al., 2011; Horstmann et al., 2011; Koritzky et al., 2012). The precise relationship between impulsivity, BMI and gender thus is not clear from previous data. Furthermore, women are more sensitive to both reward and punishment compared to men (Carver and White, 1994; Jorm et al., 1999; Cross et al., 2011). Yet, the relationship of these measures to weight status has not been sufficiently explored in males, as described earlier. Differences in the hormonal repertoire between men and women might account for variations in the susceptibility to reinforcers like food. Ovarian hormones in particular, which affect mesolimbic DA system (i.e., reward processing; Sofuoglu et al., 1999; Kaasinen et al., 2001; Evans et al., 2002; Lynch et al., 2002; Carroll et al., 2004) but also HPA functioning (i.e., stress response; Burgess and Handa, 1992; Handa et al., 1994; Patchev et al., 1995; Young, 1995), might be responsible for such differences, making women generally more vulnerable to the reinforcing properties of most drugs of abuse (see Fattore et al., 2008, 2009 for review). As addiction and obesity share several properties (see Volkow et al., 2013 for review), there might be also gender differences in the susceptibility to the reinforcing value of food. For other personality domains and their association with weight status, the gender interaction has already been shown. In a study by Faith et al. (2001) BMI was positively associated with neuroticism and negatively with extraversion in women. In men, BMI was positively associated with extraversion and psychoticism (Faith et al., 2001). Finally, gender moderates obesity-related differences in brain structure. Specifically for women obesity-related variation were observed in regions involved in habitual and goal-directed control of behavior such as the dorsal striatum and dorsolateral prefrontal cortex (Horstmann et al., 2011).

Therapeutic approaches to obesity classically target aspects of eating behavior. Behavioral interventions, for example, aim at increasing *cognitive restraint* and decreasing *disinhibition* (e.g., Jubbin and Rajesh, 2012). Yet, as described above, individual body weight status is also related to personality traits. For a more effective treatment of obesity it is therefore necessary to regard personality traits as well. This study aims to establish a comprehensive model relating BMI to eating behavior and the most relevant obesity-related personality traits (self-reported impulsivity and reward/punishment sensitivity). We investigated questionnaire measures of these traits as they can be easily and quickly assessed in the clinical setting. *TFEQ* scales *cognitive restraint*, *disinhibition*, and *hunger* (Stunkard and Messick, 1985) served as measures of eating behavior. The *BIS/BAS* scales (Carver and White, 1994) were considered as measures of sensitivity to punishment (*BIS*) and sensitivity to reward (*BAS*). Further, self-reported impulsivity, assessed by the *BIS-11* (Patton et al., 1995), was incorporated into the model. The overall goal of our approach was to quantify the individual and joint contribution of these scales to BMI variance explanation.

Based on previous findings, different models were developed to test the following hypotheses:

(1) A significant proportion of BMI variance is explained by *disinhibition*, *hunger*, and *cognitive restraint*. According to previous findings, we assumed positive linear associations of both *disinhibition* and *hunger* with BMI (e.g., Bond et al., 2001; Boschi et al., 2001; Bellisle et al., 2004; Bryant et al., 2008; Lesdéma et al., 2012). As *cognitive restraint* and BMI are positively associated in normal weight individuals and negatively in overweight and obese individuals (e.g., Foster et al., 1998; Lluch et al., 2000; Bellisle et al., 2004; Provencher et al., 2004; de Lauzon-Guillain et al., 2006; Cappelleri et al., 2009), we expected an inverted U-shaped relationship between these variables.
(2) A portion of BMI variance is explained by the interaction of *disinhibition* and *cognitive restraint*, indicated by previous studies (Stunkard and Messick, 1985; Westenhoefer et al., 1990; Williamson et al., 1995; Hays et al., 2002; Dykes et al., 2004).
(3) Additional BMI variance is explained by the level of *BIS* (as a measure of punishment responsiveness) and *BAS* (as a measure of reward responsiveness). Based on previous research, we expected positive linear associations for both variables with BMI in women (Davis et al., 2004, 2007; Franken and Muris, 2005; Harrison et al., 2010, 2011). Despite the lack of previous data for these relationships in men, we expect the positive relationships between *BIS/BAS* and BMI to be specific for women, which is based on gender-dependent differences in the hormonal repertoire influencing the vulnerability to reinforcers (e.g., Sofuoglu et al., 1999; Kaasinen et al., 2001; Evans et al., 2002; Lynch et al., 2002; Carroll et al., 2004).
(4) Further, BMI variance is explained by the level of self-reported impulsivity (*BIS-11*). According to previous findings, we expected a positive linear association with BMI (e.g., Chalmers et al., 1990; Rydén et al., 2003; Mobbs et al., 2010). Considering opposing findings with respect to gender (Pignatti et al., 2006; Brogan et al., 2011; Horstmann et al., 2011; Koritzky et al., 2012), we tested for gender interactions, although they were not expected.

Besides the study’s main purpose of modeling BMI, we had two secondary objectives:

(5) *Cognitive restraint*, *disinhibition*, and body weight status mutually influence each other (e.g., Karlsson et al., 1994; Pekkarinen et al., 1996; Foster et al., 1998; Westerterp-Plantenga et al., 1998; Dalle Grave et al., 2009; Savage et al., 2009; Tucker and Bates, 2009). Therefore, we hypothesized the quadratic relationship between BMI and *cognitive restraint* to be moderated by *disinhibition*. Depending on the level of *disinhibition*, we expected the association of BMI and *cognitive restraint* to be as follows: Normal body weight and low *disinhibition* is associated with low *cognitive restraint*. Normal body weight and high *disinhibition* is associated with high *cognitive restraint*. Overweight is associated with high *cognitive restraint* regardless of the level of *disinhibition*. Obesity is associated with low *cognitive restraint* regardless of the level of *disinhibition.*
(6) Davis and Fox (2008) demonstrated an inverted U-shaped relationship between sensitivity to reward and BMI. We aimed to corroborate these findings by testing for a quadratic relationship between *BAS* and BMI. We hypothesized an inverted U-shaped relationship between these measures.

As the focus of this investigation was on self-report questionnaires, i.e., explicit, mentally represented data, this study did not consider implicit or automatic processes (i.e., eating habits) that influence behavior and potentially body weight independently of explicit experience (e.g., Berridge and Robinson, 2003; Finlayson et al., 2008; Papies et al., 2009; Goldstein et al., 2014).

## MATERIALS AND METHODS

### SUBJECTS

Data were collected by the joint obesity work group of the Max Planck Institute for Human Cognitive and Brain Sciences and the IFB Adiposity Diseases in Leipzig between 2009 and 2013. Healthy adult subjects were invited to participate in different behavioral and neurocognitive experiments in the context of obesity research and were reimbursed for their participation. As part of these experiments, subjects completed various questionnaires this cross-sectional study is based on. Exclusion criteria were age under 18 or over 50 years, BMI under 18 kg/m^2^, hypertension, dyslipidemia, metabolic syndrome, depression (Beck’s Depression Inventory, cut-off value 18), a history of neuropsychiatric diseases, smoking, diabetes mellitus, vegetarianism, and pregnancy. Although there were no restrictions for ethnicity, only Caucasian subjects volunteered. Age in years and BMI were assessed at the time of the experiment. Height and weight for BMI calculations were measured by scientific staff at the Max Planck Institute in Leipzig. As not all questionnaires were assessed for all participants, we decided to investigate two cohorts (called *TFEQ-only* and *TFEQ-plus* cohort). The total cohort consisted of 326 healthy subjects (*TFEQ-only* cohort; 145 women, 181 men). Besides BMI, age, and gender, the *TFEQ* scores of *CR*, *DIS*, and *HUN* were assessed in these subjects. In a subgroup of 192 participants, *BIS*, *BAS*, and *BIS-11* were additionally assessed (*TFEQ-plus* cohort; 92 women, 110 men). **Table 1** depicts descriptive statistics of the two cohorts. The study was carried out in accordance with the Declaration of Helsinki and approved by the local ethics committee of the University of Leipzig. All subjects gave written informed consent before participation.

**Table 1:** Descriptive statistics.

Variable	*n*	Mean (SD)	Range	Mean women (SD)	Mean men (SD)
BMI	326	26.6 (6.1)	18.1–46.5	26.4 (6.6)	26.7 (5.6)
	192	26.7 (6.2)	18.1–46.5	26.6 (6.5)	26.8 (6.0)
Age	326	26.7 (4.8)	18–46	26.3 (4.8)	27.0 (4.9)
	192	26.6 (4.7)	18–46	25.7 (4.1)	27.2 (5.0)
CR	326	6.5 (4.6)	0–19	7.3 (5.0)	5.8 (4.1)
	192	6.7 (4.7)	0–19	7.4 (5.0)	6.2 (4.4)
DIS	326	6.1 (3.2)	0–15	6.8 (3.5)	5.6 (2.8)
	192	6.1 (3.0)	1–14	6.8 (3.3)	5.6 (2.6)
HUN	326	5.5 (3.3)	0–14	5.6 (3.3)	5.5 (3.3)
	192	5.6 (3.3)	0–14	5.9 (3.4)	5.4 (3.3)
BAS	192	30.9 (8.8)	13–51	29.7 (8.5)	31.8 (9.0)
BIS	192	17.0 (3.9)	5–26	16.5 (4.3)	17.4 (3.4)
BIS-11	192	32.2 (8.7)	9–58	32.0 (8.8)	32.3 (8.6)

*Descriptive statistics of variables assessed in the TFEQ-only cohort (*n* = 326, 145 women, 181 men) and the TFEQ-plus cohort (subgroup of TFEQ-only cohort (grey), *n* = 192, 82 women, 110 men). CR, TFEQ cognitive restraint score; DIS, TFEQ disinhibition score; HUN, TFEQ hunger score; BIS-11, Barratt Impulsiveness Scale 11 total score; BAS, Behavioral Activation System total score; BIS, Behavioral Inhibition System total score; TFEQ, Three-Factor Eating Questionnaire.*

### QUESTIONNAIRES

#### Three-Factor Eating Questionnaire (Stunkard and Messick, 1985; German version: Pudel and Westenhoefer, 1989)

The TFEQ is a 51-item self-report assessment of eating behavior. The questionnaire contains three subscales. The 21-item *cognitive restraint* scale (*CR*, scale range: 0–21, Cronbachs Alpha of German version = 0.84) measures intent to control food intake. The 16-item *disinhibition* scale (*DIS*, scale range: 0–16, Cronbachs Alpha of German version = 0.75) quantifies overeating tendencies. The 14-item susceptibility to *hunger* scale (*HUN*, scale range: 0–14, Cronbachs Alpha of German version = 0.76) is a measure for food intake in response to feelings of hunger.

#### The Behavioral Inhibition System/Behavioral Activation System Scales (Carver and White, 1994; German version: Strobel et al., 2001)

This self-report questionnaire consists of 20 items designed to assess the responsiveness of Gray’s (1982, 1987) BAS and BIS as personality characteristics. The 7-item *BIS* scale measures reactivity of the aversive motivational system (scale range: 7–28, Cronbachs Alpha of German version = 0.78), whereas the 13-item *BAS* scale measures reactivity of the appetitive motivational system (scale range: 13–52, Cronbachs Alpha of German version = 0.81). The *BAS* scale can be divided into three subscales: Drive, Fun-Seeking, and Reward. In this study we applied the *BAS* sum score, as the subscales were not confirmed in the German version.

#### Barratt Impulsiveness Scale-11 (Patton et al., 1995; German version: Preuss et al., 2008)

The *BIS-11* is a 30-item self-report questionnaire developed to measure impulsivity. Along a four-point scale subjects rate whether statements describing impulsivity pertain to themselves (scale range: 0–90, Cronbachs Alpha of German version = 0.69). For the original English version, six factors were identified. This originally suggested factor structure was not confirmed for the German equivalent. We therefore applied the total score of the *BIS-11*, as it shows adequate internal consistency for German-speaking regions.

### STATISTICAL ANALYSES

Statistical analyses were performed using SPSS (IBM Corporation Released 2011. IBM SPSS Statistics for Windows, Version 20.0. Armonk, NY: IBM Corporation) and the SPSS toolbox PROCESS (Hayes, 2013). Associations between BMI and self-reported behavioral data were explored by means of multiple regression analyses. All variables except gender were treated as continuous variables. We separately tested for the association between the three *TFEQ* scales and BMI in the *TFEQ-only* cohort (see Association of the TFEQ Scales with BMI). Age and gender were included as covariates. Significant terms were subsequently used to build a regression model for BMI to assess the proportion of variance solely explained by variables of eating behavior (see BMI Modeling Based on the TFEQ Scales Cognitive Restraint and Disinhibition). Next, we tested *BIS-11*, *BIS*, and *BAS* seperately for their association with BMI in the *TFEQ-plus* cohort (see Association of the Barratt Impulsiveness Scale-11, Behavioral Activation System, and Behavioral Inhibition System Scales with BMI). Additionally, gender interactions for the relationships of the latter three scores with BMI were tested. Age and gender were included as covariates. Again, all significant terms were used to build a comprehensive regression model for BMI including eating behavior and personality traits (see BMI Modeling Based on Cognitive Restraint, Disinhibition, the Behavioral Activation System, and Behavioral Inhibition System Score).

Based on findings of previous studies, quadratic relationships between BMI and *CR* (moderated by *DIS*, see Interactions between Cognitive Restraint, Disinhibition, and BMI) and between BMI and *BAS* (see Quadratic Relationship between BMI and the Behavioral Activation System Score) were tested (Foster et al., 1998; Lluch et al., 2000; Bellisle et al., 2004; Provencher et al., 2004; de Lauzon-Guillain et al., 2006; Davis and Fox, 2008; Cappelleri et al., 2009). BMI was treated as regressor for these analyses.

**Table 2** lists the regression models which were used to test all abovementioned associations. As measures of effect size we used partial correlations and squared partial correlations. The latter can be interpreted as the regressand’s (e.g., BMI) proportion of variance which can be explained by a single regressor (e.g., *DIS*) when all other variables are held constant. For reasons of consistency, not to indicate causality, BMI was depicted at the x-axis of every graph. We added a table of Pearson Correlations of the assessed variables at the end of the results section (see Pearson Correlations of All Variables of Interest).

**Table 2:** Regression models and corresponding variables.

Association with regressand	Variables in model	Tested gender interaction
Linear	A, g, a	A*g
Quadratic (e.g., CR^2^)	A, A^2^, g, a	A^2^*g
2-way interaction (DIS*CR)	A, B, A*B, g, a	–
Quadratic 2-way interaction (BMI^2^*DIS)	A, B, A^2^, A*B, A^2^*B, g, a	–

*Different regression models were computed to test our individual hypotheses. Corresponding variables of all the investigated models are listed. Partial correlations of the underlined terms were tested against 0. A, B: tested variables, e.g., Three-Factor Eating Questionnaire cognitive restraint (CR) or disinhibition score (DIS); g, gender; a, age.*

## RESULTS

### TFEQ-ONLY COHORT (*n* = 326)

#### Association of the Three-Factor Eating Questionnaire scales with BMI

**FIGURE 1:**
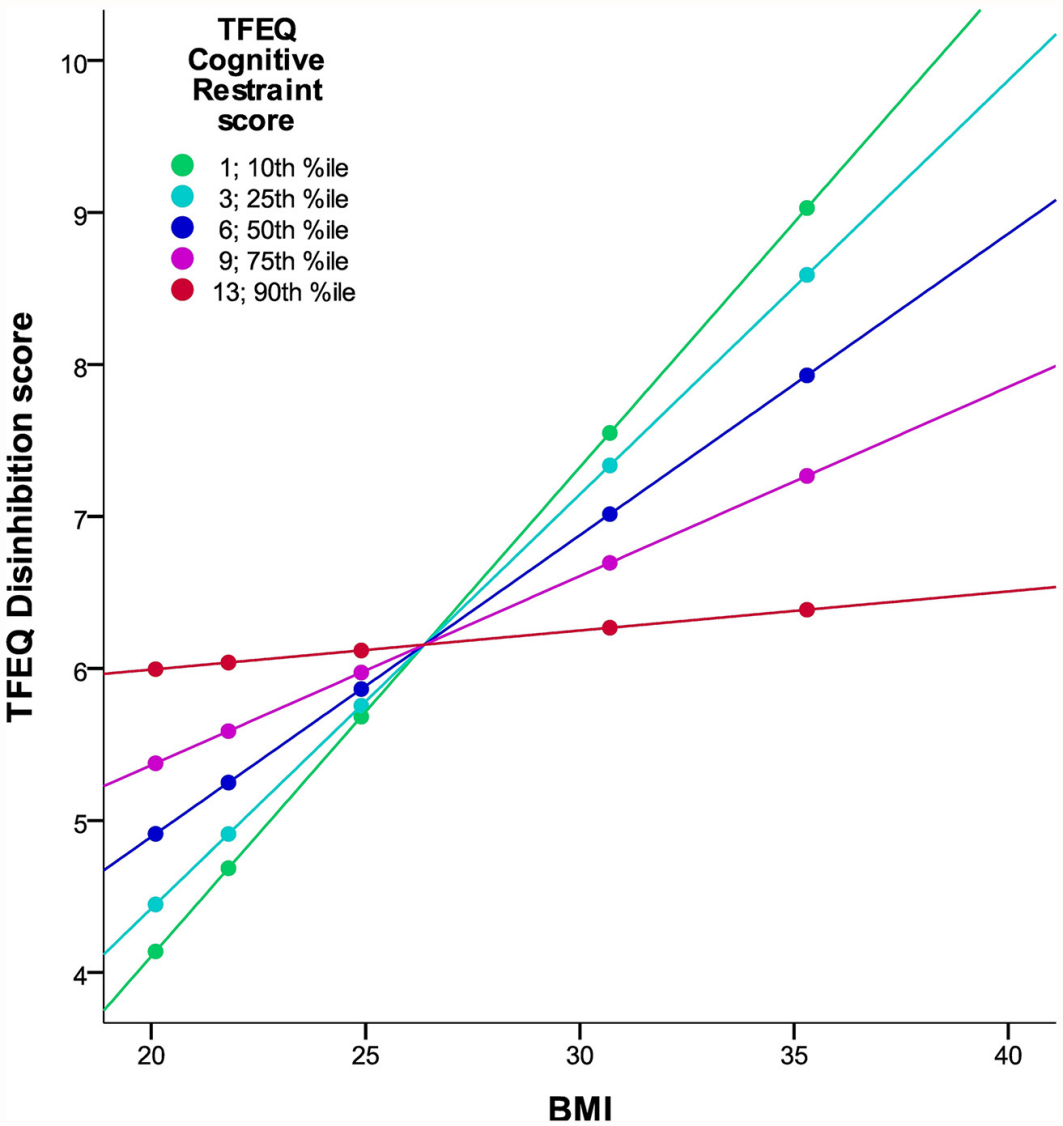
**Interaction of DIS and CR on BMI in the TFEQ-only cohort (*n*= 326).** The figure illustrates the linear relationship between BMI and DIS moderated by the level of CR with age and gender as covariates. Partial correlation of BMI*CR is -0.203 (*p* < 0.0005; adjusted *R*^2^ change of 0.163 through BMI, CR, and BMI*CR). Dots indicate 10th, 25th, 50th, 75th, and 90th percentiles of BMI (20.1, 21.8, 24.9, 30.7, and 35.3 kg/m^2^). Colors indicate 10th, 25th, 50th, 75th, and 90th percentiles of CR (1, 3, 6, 9, 13). CR, cognitive restraint score; DIS, disinhibition score; TFEQ, Three-Factor Eating Questionnaire.

In the total cohort of 326 subjects, a gender difference in *CR* (*p* = 0.004) and in *DIS* (*p* = 0.001) was observed, with women having higher scores in both cases. BMI significantly correlated with *DIS*, *CR*^2^ (hypothesis 1), and the interaction term of *CR* and *DIS* (hypothesis 2; **Figure 1**; partial correlations, all *p* < 0.0005; see **Table 3**). We observed no significant association of *HUN* with BMI.

**Table 3:** Squared partial correlations (SPC) with BMI.

Variable	Squared partial correlation ($\eta_{p}^{2}$)	Direction of correlation	*p*-value
CR	(0.009)	(+)	0.083
DIS	0.138	+	<0.0005
HUN	(0.003)	(+)	0.596
CR^2^	0.054	-	<0.0005
CR*DIS	0.054	-	<0.0005

*Squared partial correlations with BMI in the TFEQ-only cohort (*n*= 326) in a regression model with age and gender as covariates. SPC can be interpreted as the proportion of BMI variance explained only by the corresponding variable, not by covariables. CR, TFEQ cognitive restraint score; DIS, TFEQ disinhibition score; HUN, TFEQ hunger score; TFEQ, Three-Factor Eating Questionnaire.*

#### BMI modeling based on the *TFEQ* scales *cognitive restraint* and *disinhibition*

To obtain a model for BMI regressed on the *TFEQ* scales, a multiple regression analysis using all former significant terms (i.e., *CR*, *DIS*, *CR*^2^, and *CR*^∗^*DIS*; additional covariates age and gender) was conducted. The underlying adjusted *R*^2^ of this model was 0.232 (women: 0.247, men: 0.208). *CR*^∗^*DIS* as well as *CR*^2^ separately explained part of BMI variance, as their partial correlations differed from 0 (both *p* < 0.0005). Hence, the *TFEQ* scales *CR* and *DIS* (in addition to age and gender) explained about 23% of the overall variance of BMI in the population of this cohort.

#### Interactions between cognitive restraint, disinhibition, and BMI

**FIGURE 2:**
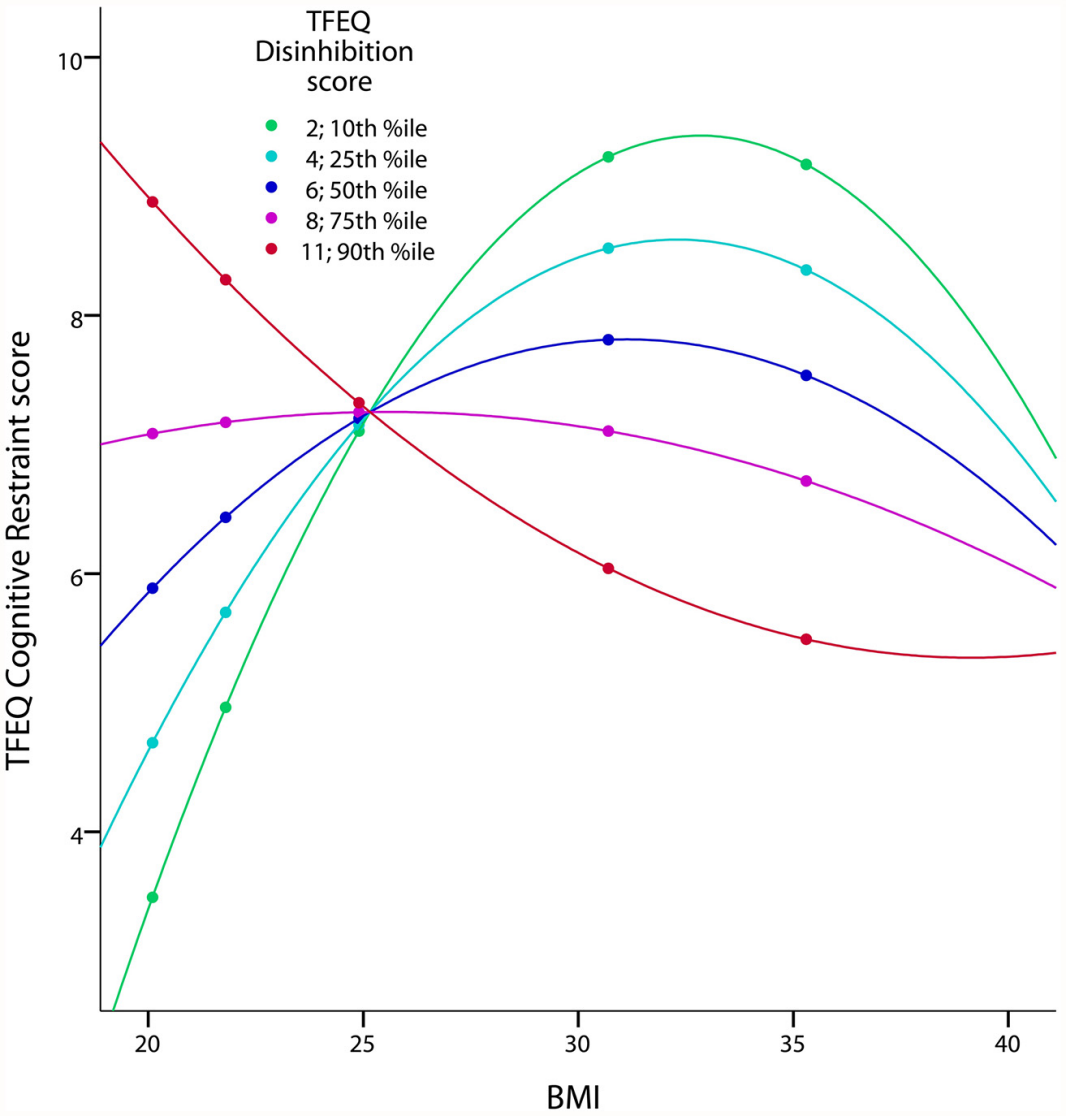
**Quadratic interaction of BMI and DIS on CR in the TFEQ-only cohort (*n*= 326).** The figure illustrates the quadratic relationship between BMI and CR moderated by the level of DIS with age and gender as covariates. Partial correlation of BMI^2^*DIS is 0.185 (*p*< 0.001; adjusted *R*^2^ change of 0.083 through BMI, DIS, BMI^2^, BMI*DIS and BMI^2^*DIS). Dots indicate 10th, 25th, 50th, 75th, and 90th percentile of BMI (20.1, 21.8, 24.9, 30.7, and 35.3 kg/m^2^). Colors indicate10th, 25th, 50th, 75th, and 90th percentiles of CR (2, 4, 6, 8, 10). CR, cognitive restraint score; DIS, disinhibition score; TFEQ, Three-Factor Eating Questionnaire.

We hypothesized a quadratic relationship between *CR* and BMI (hypothesis 5). The regression of *CR* on *BMI*^2^ confirmed this hypothesis (squared partial correlation: 0.029, *p*= 0.002, age and gender as covariates). Furthermore, this inverted U-shaped relationship was moderated by *DIS* (*p*= 0.001). In other words, the relationship between BMI and *CR* differed with respect to the *DIS* score (**Figure 2**): For low *DIS* scores the quadratic association between *CR* and BMI was well pronounced, whereas no strong quadratic relationship for high *DIS* scores was observed.

### TFEQ-PLUS COHORT (*n* = 192)

#### Association of the Barratt Impulsiveness Scale-11, Behavioral Activation System, and Behavioral Inhibition System Scales with BMI

With respect to eating behavior (based on the *TFEQ*), results in the subgroup of 192 participants (*TFEQ-plus* cohort) were comparable with the whole sample (*TFEQ-only* cohort, *n*= 326).

**FIGURE 3:**
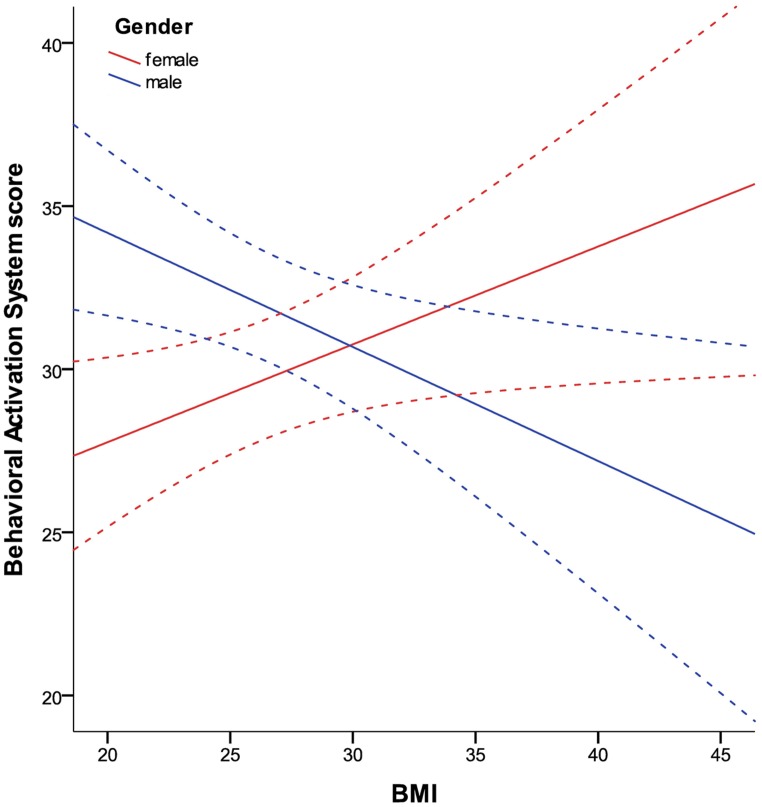
**Relationship between BMI and BAS in women and men in the TFEQ-plus cohort (*n*= 192).** As the relationship of BAS and BMI is moderated by gender, it is shown separately. Partial correlation of BMI*gender is -0.255 (*p*< 0.0005, age as covariate). Partial correlation of BMI (age as covariate) with BAS is 0.214 in women (*n*= 82) and -0.295 in men (*n*= 110). Dashed lines indicate confidence interval of 95% for the fit lines. BAS, Behavioral Activation System total score.

*BAS* and *BIS* scores did not correlate with BMI, but showed a significant interaction with gender (hypothesis 3; all *p*= 0.001). In women, there was a significant positive correlation of *BIS* and BMI (partial correlation = 0.281; *p*= 0.011) as well as a strong tendency for the correlation of *BAS* and BMI (partial correlation = 0.214; *p*= 0.055). In men, we found a significant negative correlation of *BIS* and BMI (partial correlation = -0.208; *p*= 0.03) as well as *BAS* and BMI (partial correlation = -0.295; *p*= 0.002). The relationship of BMI and *BAS*, moderated by gender, is shown in **Figure 3** (results for the association of *BIS* and BMI are comparable). Concerning the association of self-reported impulsivity and BMI, neither a correlation between BMI and *BIS-11* (total score) nor a gender interaction was found (hypothesis 4).

#### BMI modeling based on cognitive restraint, disinhibition, the Behavioral Activation System, and Behavioral Inhibition System score

**FIGURE 4:**
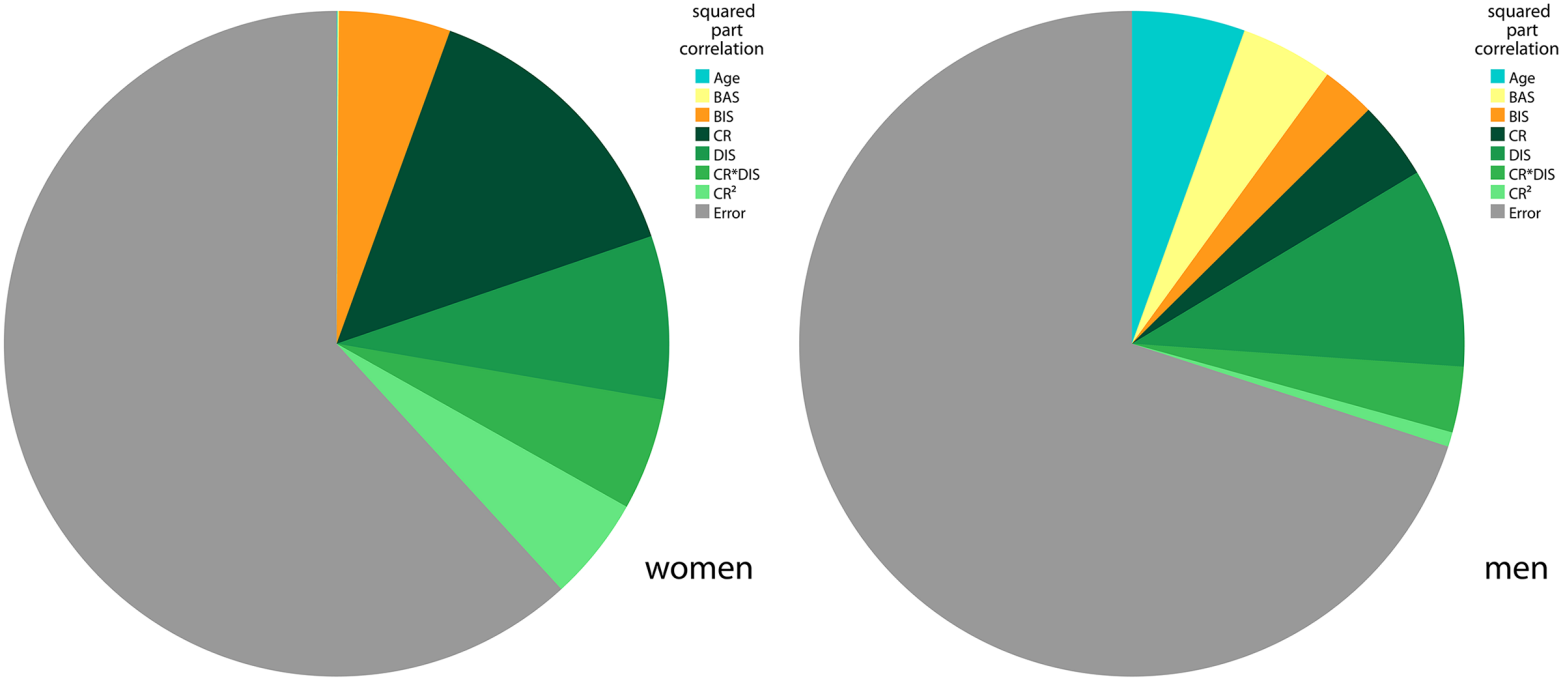
**BMI variance explained by final regression model in men and women.** The pie charts show the squared part correlations of all variables of the final BMI model in the TFEQ-plus cohort (*n*= 192). All variables with significant correlation to BMI were included. As the directions of the effect of BAS and BIS differed between men and women, separate models comprising the same variables were computed. *R*^2^ for women (*n*= 82) = 0.382. *R*^2^ for men (*n*= 110) = 0.300. CR, TFEQ cognitive restraint score; DIS, TFEQ disinhibition score; BAS, Behavioral Activation System total score; BIS, Behavioral Inhibition System total score; TFEQ, Three-Factor Eating Questionnaire.

The final model comprised the relevant variables of self-reported eating behavior (see BMI Modeling based on the TFEQ Scales Cognitive Restraint and Disinhibition, *TFEQ-only* model) as well as *BIS*, *BAS*, gender, *BIS*^∗^gender, *BAS*^∗^gender and age as regressors. The resulting adjusted *R*^2^ was 0.271 (women: 0.324, men: 0.252). *R*^2^ for women and men did not differ significantly (*p* = 0.474, two-tailed Fisher’s Z). Independent of eating behavior, *BIS* and *BAS* significantly contributed to variance explanation of BMI (*R*^2^ change of *TFEQ-only* model and *TFEQ-plus* model in the sample of *n*= 192, *p* < 0.0005). Hence, self-reported behavioral measures of *CR*, *DIS*, *BIS*, and *BAS* in addition to age and gender explained about 27% of the overall variance of BMI in the population of this sample. See **Figure 4** for variance proportions of the variables for each gender.

#### Quadratic relationship between BMI and the Behavioral Activation System score

**FIGURE 5:**
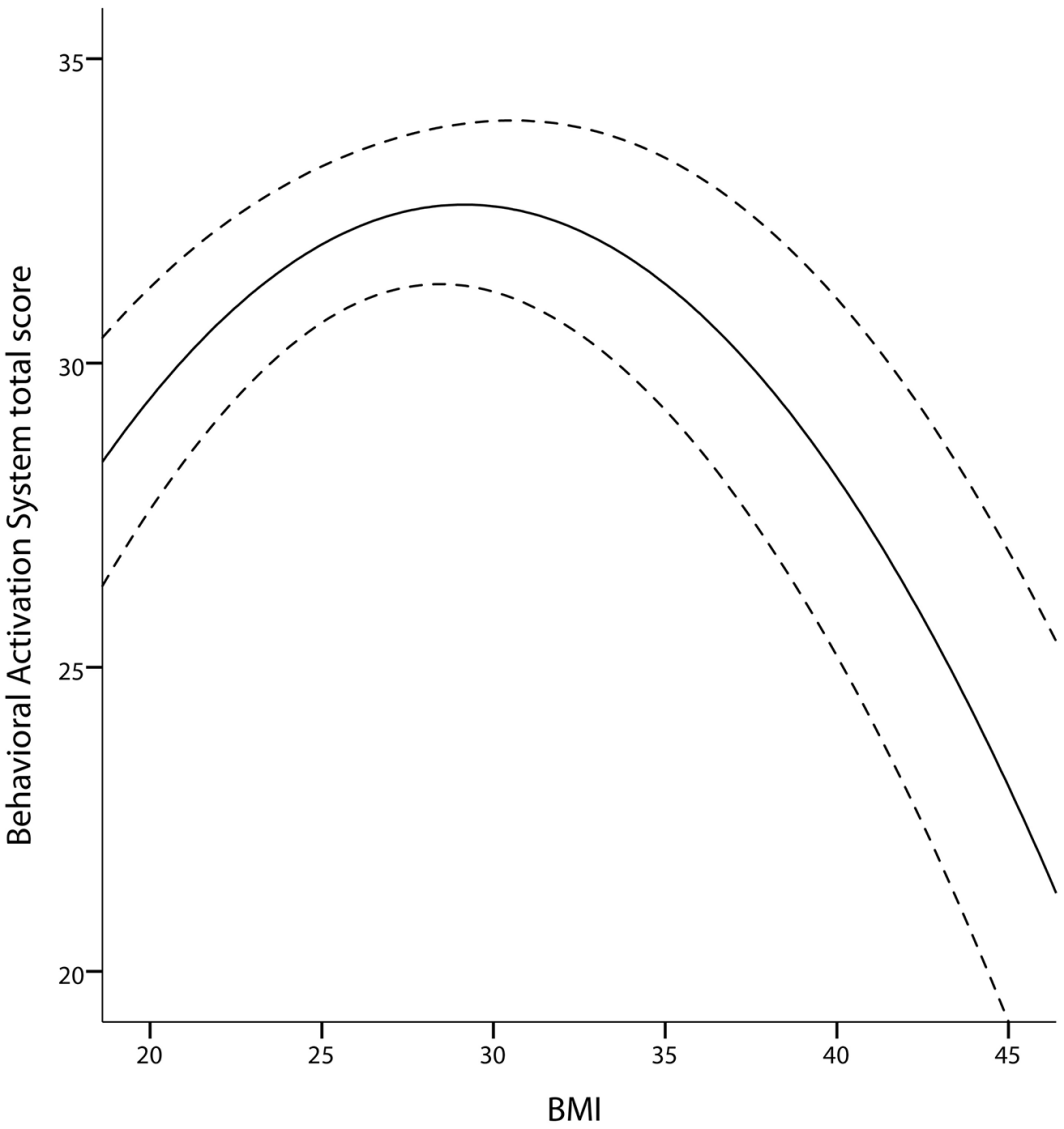
**Quadratic association between BAS and BMI in the TFEQ-plus cohort (*n*= 192).** Partial correlation of BMI^2^ is -0.92 (*p*= 0.008, adjusted *R*^2^ change of 0.039 through BMI and BMI^2^, age and gender as covariates). Dashed lines indicate the 95% confidence interval of the quadratic fit line. BAS, Behavioral Activation System total score.

As Davis and Fox (2008) reported an inverted U-shaped association between sensitivity to reward and BMI, we tested for the quadratic association of *BAS* with BMI (hypothesis 6). We corroborated their finding: BMI showed a quadratic relationship with *BAS* (*p*= 0.018, age and gender as covariates, adjusted *R*^2^ changed by 0.03 after adding BMI and BMI^2^). There was only a trend for a gender interaction of this effect (*p*= 0.091, stronger effect in women). Concerning the model, a BMI of around 30 kg/m^2^ was associated with the highest *BAS* scores, whereas a higher and lower BMI was associated with lower *BAS* scores (**Figure 5**).

### PEARSON CORRELATIONS OF ALL VARIABLES OF INTEREST

For an overview of the assessed variables and how they are interrelated, see **Table 4**. As the correlation of *BIS* and *BAS* was not described thus far, this association was further investigated. One reason for this relationship might be the high proportion of obese subjects in our sample. Therefore we tested for an interaction of BMI with *BIS* or *BAS*. Also gender interactions of this assumed effects were tested. We found a 3-way-interaction between BMI, gender and *BIS* (*p*= 0.007 for *BIS*^∗^BMI^∗^gender with *BAS* as regressand; age as covariate). Probing this 3-way-interaction revealed that women with a high BMI had a stronger association of *BIS* with *BAS*.

**Table 4:** Pearson correlations.

		BIS-11	BAS	BIS	HUN	DIS
**CR**	*r*	-0.196**	0.180*	0.018	-0.227***	0.148*
	*p*	0.006	0.013	0.801	**0.002**	0.041
**DIS**	*r*	0.046	0.135	-0.022	0.494***	
	*p*	0.525	0.061	0.764	**< 0.0005**	
**HUN**	*r*	0.195**	-0.050	-0.062		
	*p*	0.007	0.487	0.391		
**BIS**	*r*	-0.002	0.324***			
	*p*	0.981	**< 0.0005**			
**BAS**	*r*	-0.132				
	*p*	0.068				

*p < 0.05, **p < 0.01, ***p < 0.0033.

Pearson correlations between all assessed questionnaire scores in the TFEQ-plus cohort (*n*= 192). *p*-values < 0.0033 (***, bold) are considered as significant after Bonferroni correction for multiple comparison. Noticeable are the associations of CR with HUN (negative), DIS with HUN (positive), and BAS with BIS (positive) as well as the trend toward the correlation of CR and BIS-11 (negative). CR, TFEQ cognitive restraint score; DIS, TFEQ disinhibition score; HUN, TFEQ hunger score; BIS-11, Barratt Impulsiveness Scale 11 total score; BAS, Behavioral Activation System total score; BIS, Behavioral Inhibition System total score; TFEQ, Three-Factor Eating Questionnaire.

## DISCUSSION

### RELATIONSHIP BETWEEN EATING BEHAVIOR AND BMI

Interestingly, only two measures of eating behavior, *disinhibition* and *cognitive restraint*, accounted for much of BMI variance (∼23%). In other words, the individual level of overeating tendencies in interaction with the level of conscious efforts to restrict food intake explained a large amount of variance in individual body weight status. *Susceptibility to hunger* did not contribute to variance explanation of BMI. However, an association of *hunger* with *disinhibition* and *cognitive restraint* was shown in our sample, which is in line with previous studies (Bellisle et al., 2004; Lesdéma et al., 2012).

Besides modeling of BMI, we aimed to investigate the apparent non-linear relationship between *cognitive restraint* and BMI. We found an inverted U-shaped association of BMI with *cognitive restraint*. Our model demonstrates low levels of *cognitive restraint* at the outer edges of the BMI range and a high level around the overweight range. Interestingly, this relationship was moderated by the level of *disinhibition*. For low levels of *disinhibition* (low overeating tendencies) the curvilinear relationship between BMI and *cognitive restraint* was well pronounced. Accordingly, we conclude that restrained eating is low in normal weight individuals as food restriction is presumably not necessary. With higher BMI, food restriction becomes necessary, as losing weight or avoiding further weight gain are supposedly more frequent with higher BMI (maximum in the overweight/moderate obese range of the BMI). In the obese BMI range, the positive relationship between BMI and *cognitive restraint* is shifted, resulting in relatively low levels of restrained eating among morbidly obese individuals. Although restrained eating seems desirable in this BMI range, morbidly obese individuals might not be able to raise sufficient self-control resources to restrict food intake. This notion is supported by neuroimaging studies that report structural as well as functional obesity-related alterations in brain structures associated with self-control (Le et al., 2006, 2007; Horstmann et al., 2011). With higher levels of *disinhibition* there was no strong curvilinear relationship between BMI and *cognitive restraint*. This effect indicates that in response to heightened overeating tendencies, normal weight individuals increase conscious efforts to restrict food intake in order to maintain weight/stay slim. Overweight and moderately obese individuals presumably do not adequately adapt their dietary restraint. On the contrary, the model indicates that attempts to restrict food intake decrease (reflected in lower levels of *cognitive restraint*) with stronger disinhibited eating. Eating behavior seems to be more and more dominated by an uncontrolled eating style, driven by, for example, external eating signals or habitual food intake.

### GENDER-SPECIFIC RELATIONSHIPS BETWEEN *BIS/BAS* AND BMI

The aforementioned model for BMI based on eating behavior was extended to incorporate personality factors not inherently related to food context but potentially influencing body weight. Both *BIS* and *BAS* explained part of BMI variance independently of eating behavior (∼6%), whereby they inversely accounted for BMI variance in men and women. Both scales were positively associated with BMI in women, but negatively in men.

### *BAS* RESPONSIVENESS AND BMI

Studies already showed that reward responsiveness is positively related to body weight status and eating habits contributing to weight gain in women (Davis and Woodside, 2002; Davis et al., 2004; Franken and Muris, 2005; Loxton and Dawe, 2006). Women report more food cravings than men, indicating heightened motivation for hedonic eating (Lafay et al., 2001; Cepeda-Benito et al., 2003; Meule et al., 2012). Moreover, several studies have shown that women are highly susceptible to the sociocultural pressure resulting from the “lean ideal” portrayed by the media, leading to attempts to lose weight and be slim (Polivy and Herman, 2004; Dittmar, 2005; Mask and Blanchard, 2011; Yean et al., 2013). As a consequence food restriction and avoidance behavior might boost initial vulnerability to and incentive saliency of highly palatable “forbidden” food. In males, drive for a lean body has been shown to be lower (e.g., Cohane and Pope, 2001; Grogan and Richards, 2002; Yean et al., 2013). Their individual motivational value of food might thus be less environmentally influenced. For men, reward associated with novelty and excitement might be particularly reinforcing. Studies reported a higher risk for excitement-related addiction like pathological gambling (see van den Bos et al., 2013a for review), alcohol and cannabis (Wagner and Anthony, 2007; NSDUH, 2012; EMCDAA, 2013) or exercise dependence (Crossman et al., 1987; Pierce et al., 1997; Weik and Hale, 2009) in men.

### *BIS* RESPONSIVENESS AND BMI

Emotional eating, which is related to punishment sensitivity (Gray, 1970, 1982, 1987), serves as a way to compensate perceived punishment/negative affect in women (van Strien et al., 1986, 2013; Geliebter and Aversa, 2003; Nolan, 2012). Therefore obesity in women with high *BIS* responsiveness might be related to compensational eating. Men generally show a lower sensitivity to punishment (Cross et al., 2011) as well as stronger emotional and cognitive control over immediate emotional events (especially punishments; van den Bos et al., 2013b), presumably reducing their need for compensation of negative emotionality. Further, there is no clear-cut link between negative emotional eating and BMI in men (Macht et al., 2002; Geliebter and Aversa, 2003; Nolan, 2012), and, in contrast to women, food craving has been associated with positive mood states (Lafay et al., 2001). In contrast to women *BIS* responsiveness in men might reflect differences in risk taking behavior. Koritzky et al. (2012) showed that particularly overweight and obese in comparison to lean men decided more often for high immediate reward despite long-term losses. Accordingly, they might more easily ignore long-term consequences of overeating, such as weight gain, because of low sensitivity to related punishment.

Although the *BIS* and *BAS* scales are assumed to be orthogonal (Gray, 1982, 1987), we found a correlation between the two measures. As BMI moderated the relationship between *BIS* and *BAS* in women, we assume that differences in body weight status accounted for this effect in our sample.

### INVERTED U-SHAPED RELATIONSHIP BETWEEN BMI AND *BAS*

We corroborated the inverted U-shaped relationship between sensitivity to reward and BMI demonstrated by Davis and Fox (2008) using the *BAS* scale. Following Davis and Fox (2008), subjects with a high BMI in the non-obese range are supposed to face stronger food cravings and appetitive drive, resulting in enhanced hedonic eating, weight gain, and possibly overweight. Davis and Fox (2008) assumed that these individuals detect rewarding stimuli like palatable food more easily and more likely approach them. The inverse relationship between BMI and *BAS* in the obese range of the BMI is supposed to reflect reward deficiency resulting from hypo-DA functioning in obese individuals (Wang et al., 2001; Volkow et al., 2008; de Weijer et al., 2011). Compensatory hedonic eating probably compensate for this deficiency.

### RELATIONSHIP BETWEEN SELF-REPORTED IMPULSIVITY AND BMI

The contribution of self-reported impulsivity on body weight remains vague. Impulsivity did not explain BMI variance in our dataset. Contradictory results regarding the relationship with BMI have been reported previously (Nolan, 2012; van Koningsbruggen et al., 2013). In general, none of the subscales seem to be consistently related to overeating or BMI (Meule, 2013). However, we observed a trend for a negative correlation between *BIS-11* and *cognitive restraint*. This indicates an indirect influence of impulsivity on body weight status via eating behavior, which is in line with previous findings (Leitch et al., 2013).

### STUDY LIMITATIONS AND FUTURE DIRECTIONS

This study is based on analyses of self-reported measures, i.e., mentally represented, explicitly accessible information. We have not considered automatic processes (i.e., eating habits) like implicit food attitudes (e.g., Papies et al., 2009; Goldstein et al., 2014) or implicit liking/wanting (e.g., Berridge and Robinson, 2003; Finlayson et al., 2008), which should be regarded in future studies.

Furthermore, impulsivity is a multifaceted construct (e.g., Patton et al., 1995; Whiteside and Lynam, 2001). According to insufficient validity of the factor structure of the *BIS-11* in German (Preuss et al., 2008) we restricted our analysis to the *BIS-11* total score. Another impulsivity scale, the *UPPS Impulsive Behavior Scale* (Whiteside and Lynam, 2001), is recommended as an additional self-report measure of impulsivity. This scale is associated with obesity (Mobbs et al., 2010), but probably measures aspects of impulsivity that are not covered by *BIS-11* (Meule, 2013).

Moreover, *cognitive restraint* has been proposed to be subdivided into a rigid and flexible component (Westenhoefer, 1991; Westenhoefer et al., 1999). For reasons of construct validity, the *cognitive restraint* scale has been expanded by several further items (Westenhoefer et al., 1999). We recommend assessment of these items, because subscaling allows a more detailed analysis of *cognitive restraint’s* influence on body weight.

Finally, BMI, although a common way to assess obesity, is a rather course measure. It relates body weight to body height without taking actual body composition into account. As it does not measure body fat directly, erroneous evaluation of body weight status with respect to obesity can occur (Rothman, 2008). Addressing this limitation, we recommend consideration of additional measures like waist/hip ratio or concentration of adipokines like leptin (Badman and Flier, 2005).

### SUMMARY

This study demonstrates that responsiveness to the behavioral activation and behavioral inhibition system explains differences in BMI independently of eating behavior. Interestingly the relationships of BMI to *BIS* and *BAS* depend on gender, with opposing directions in men and women. Therefore, specified for men and women, *BIS/BAS* responsiveness should be considered in the treatment of obesity. Further, our study contributes to a better understanding of the complex relationships between eating behavior and body weight status. We showed that *cognitive restraint* and BMI are non-linearly associated (inverted U-shaped relationship). Importantly, this relationship is moderated by the level of *disinhibition.*

## Conflict of Interest Statement

The authors declare that the research was conducted in the absence of any commercial or financial relationships that could be construed as a potential conflict of interest.
